# Clinical and prognostic significances of cancer stem cell markers in gastric cancer patients: a systematic review and meta-analysis

**DOI:** 10.1186/s12935-021-01840-z

**Published:** 2021-02-27

**Authors:** Mahdieh Razmi, Roya Ghods, Somayeh Vafaei, Maryam Sahlolbei, Leili Saeednejad Zanjani, Zahra Madjd

**Affiliations:** 1 Oncopathology Research Center, Iran University of Medical Sciences (IUMS), Tehran, Iran; 2 Department of Molecular Medicine, Faculty of Advanced Technologies in Medicine, Iran University of Medical Sciences, Tehran, Iran

**Keywords:** Cancer stem cells, Gastric cancer, Prognostic value, Clinicopathological characteristics, Meta-analysis

## Abstract

**Background:**

Gastric cancer (GC) is considered one of the most lethal malignancies worldwide, which is accompanied by a poor prognosis. Although reports regarding the importance of cancer stem cell (CSC) markers in gastric cancer progression have rapidly developed over the last few decades, their clinicopathological and prognostic values in gastric cancer still remain inconclusive. Therefore, the current meta-analysis aimed to quantitatively re-evaluate the association of CSC markers expression, overall and individually, with GC patients’ clinical and survival outcomes.

**Methods:**

Literature databases including PubMed, Scopus, ISI Web of Science, and Embase were searched to identify the eligible articles. Hazard ratios (HRs) or odds ratios (ORs) with 95% confidence intervals (CIs) were recorded or calculated to determine the relationships between CSC markers expression positivity and overall survival (OS), disease-free survival (DFS)/relapse-free survival (RFS), disease-specific survival (DSS)/ cancer-specific survival (CSS), and clinicopathological features.

**Results:**

We initially retrieved 4,425 articles, of which a total of 66 articles with 89 studies were considered as eligible for this meta-analysis, comprising of 11,274 GC patients. Overall data analyses indicated that the overexpression of CSC markers is associated with TNM stage (OR = 2.19, 95% CI 1.84–2.61, *P* = 0.013), lymph node metastasis (OR = 1.76, 95% CI 1.54–2.02, *P* < 0.001), worse OS (HR = 1.65, 95% CI 1.54–1.77, *P* < 0.001), poor CSS/DSS (HR = 1.69, 95% CI 1.33–2.15, *P* < 0.001), and unfavorable DFS/RFS (HR = 2.35, 95% CI 1.90–2.89, *P* < 0.001) in GC patients. However, CSC markers expression was found to be slightly linked to tumor differentiation (OR = 1.25, 95% CI 1.01–1.55, *P* = 0.035). Sub-analysis demonstrated a significant positive relationship between most of the individual markers, specially Gli-1, Oct-4, CD44, CD44V6, and CD133, and clinical outcomes as well as the reduced survival, whereas overexpression of Lgr-5, Nanog, and sonic hedgehog (Shh) was not found to be related to the majority of clinical outcomes in GC patients.

**Conclusion:**

The expression of CSC markers is mostly associated with worse outcomes in patients with GC, both overall and individual. The detection of a combined panel of CSC markers might be appropriate as a prognostic stratification marker to predict tumor aggressiveness and poor prognosis in patients with GC, which probably results in identifying novel potential targets for therapeutic approaches.

## Introduction

Gastric cancer (GC) is known as the fifth most common malignancy and the third leading cause of cancer-associated mortality worldwide [1, 2]. It has been reported that nearly 800,000 gastric cancer-related deaths occur annually, with an average 5-year survival rate of less than 30%, which geographically are more frequent in Asian, Eastern European, and South American countries [3]. Although the GC mortality rate has reduced over the last decade due to surgery, chemotherapy, and targeted therapy, the disease burden still remains high with a remarkable unsatisfactory prognosis. Moreover, a high rate of advanced-stage diagnosis, lack of appropriate predictive markers, the progression of recurrence and metastasis, and treatment failure are the key factors that contribute to the poor prognosis of patients with this disease [4].

Of note, biomarkers have become valuable promising tools for improving and optimizing diagnosis, treatment, and prognosis of GC [5]. Nevertheless, the restricted confirmation and controversial prognostic values of the current clinical biomarkers led to qualifying inadequate as robust biomarkers to be implemented in clinical practice for GC patients [6]. Therefore, the molecular pathogenesis of development and progression in GC is still unclear, and more prognosis biomarkers of GC are waiting to be uncovered.

Recently, researchers have focused on identifying and targeting cancer stem cells (CSCs). The heterogeneous phenotype of tumor is considered as a key driver of treatment resistance and cancer recurrence, for which CSCs are considered to be among the major causes of tumor heterogeneity and therapy’s failure [7]. CSCs are defined as a unique subpopulation of cancer cells that possess self-renewal and differentiation potentials, consequently deliberating cancer initiation, invasion, metastasis, relapse, and chemoresistance [8, 9].

Growing evidence supports that overexpression of multiple specific stemness genes in gastrointestinal stem cells may promote the self-renewal capacity of CSCs in GC and are linked to patients’ prognosis [10]. Several cell-surface markers, including CD133, CD44, CD166, and CD24, have been identified as gastric CSC markers [11]. In addition, some pluripotent transcription factors (TFs), including Oct-4, KLF4, MYC, Nanog, and Sox-2 and some intracellular signaling cascades, including Wnt, Sonic hedgehog (Shh), NF-κB, and Notch as well as extracellular factors, are known as essential regulators of CSCs [12]. Recently, many scholars have recognized the relationship of overexpression of CSC-related key markers and possible signal pathways with prognostic and clinical values in gastric carcinoma [13, 14]. However, as a consequence of diversities in study methodology, research participants, and sample size, there are some conflicting opinions on the gastric tumor that need to be addressed. The analysis of these markers may potentially result in the identification of some potential prognostic biomarkers and novel therapeutic targets in GC.

Therefore, we aimed to collect all available data and perform the current meta-analysis on the potential role of CSC-related biomarkers as clinical and prognostic biomarkers in GC patients in order to clarify controversial issues and explain which one of these biomarkers has more clinical importance regarding the quantitative evidence.

## Materials and methods

### Data sources and literature search strategy

Literature searches, based on the relationship between gastric CSC markers expression and clinical outcomes, were thoroughly performed from PubMed, Scopus, Embase, and ISI Web of Science databases until February 26, 2020, and updated on October 16, 2020. All the searches were restricted to English publications. The search strategy was based on the following main keywords: (neoplasm OR cancer OR tumor OR malignancy OR malignant OR carcinoma) AND (stomach OR gastric) AND (neoplastic stem cell OR neoplastic colony-forming unit OR tumor stem cell OR tumor-initiating cell OR cancer stem cell OR CSC) AND (biomarker OR marker OR prognosis OR prognostic OR diagnosis OR diagnostic OR screening OR detection). The strategy of the literature searching has been presented in Additional file 1: Table S1. The protocol for the current meta-analysis was performed in accordance with the recommendations of the Preferred Reporting Items for Systematic Reviews and Meta-Analyses (PRISMA) statement [15].

### Eligibility criteria

Studies were included based on the following criteria: (1) case–control or cohort studies published in English as original studies with available full texts; (2) studies with human gastric tumor; (3) the expression of gastric CSC-relevant markers detected by immunohistochemistry (IHC) in primary tumor tissues instead of serum or other kinds of specimens; (4) studies evaluating the association between the CSC markers expression and overall survival (OS), disease-free survival (DFS)/relapse-free survival (RFS), and disease-specific survival (DSS)/ cancer-specific survival (CSS), and/or clinicopathological features of GC; and (5) hazard ratios (HRs) with 95% confidence intervals (CIs) presented in the text, or availability of data in order to calculate HRs and 95% CIs.

The studies were excluded on the basis of the following criteria: (1) book chapters, reviews, letters, and conference abstracts; (2) studies were not related to the topic of the interest (e.g., when the studies investigated other solid tumors or other diseases); (3) in vitro and animal studies; (4) studies in which participants administered any kind of anticancer therapy such as radiotherapy and chemotherapy, prior to biopsy; and (5) studies with lack of sufficient and useful data.

### Study selection and data extraction

All search records were transferred to Endnote software to remove the duplicate files. The eligible studies were identified after the independent screening of the titles and abstracts based on the inclusion criteria by two investigators (MR and RG). Discrepancies were resolved through discussion or by a third investigator. Two independent researchers (MR and RG) extracted prognostic or clinicopathological data from eligible articles in a predefined table. For each of the included articles, the following descriptive data were collected: the name of the first author, country and year of conduction of the study, detection method, age, sex, sample size, CSC marker*,* case number of different groups, median or mean follow-up times, clinicopathological parameters, cut-off value, and the related survival data. HR and 95% CI of OS, DFS/RFS, and CSS/DSS were taken into account for counting pooled HR. Where HR was not reported, the calculation method was applied to extract HR and 95% CI. The primary outcome was the relationship between the CSC markers expression and OS, DFS/RFS, or CSS/DSS in GC patients. Other outcomes of interest were the relationships between the CSC markers expression and the important clinicopathological parameters of GC. All the extracted data were cross-checked by SV, MS, and LS.

### Quality assessment

The quality of all eligible studies was evaluated through the Newcastle***–***Ottawa Scale (NOS) [16]. Accordingly, the NOS evaluates the quality of studies based on three parameters, i.e., selection, comparability, and exposure or outcome, with a score between 0 to 9. Articles with NOS points above 6 were determined as high-quality studies. Any disagreement was discussed and then resolved by consensus.

### Statistical analysis

The associations between gastric CSC markers expression and clinicopathological characteristics, including TNM stage (III/IV vs. I/II), tumor differentiation (poor vs. well/moderate), and lymph node metastasis (positive vs. negative), were evaluated by combining the odds ratios (ORs) and 95% CIs. In the current analysis, an OR > 1 demonstrated a higher possibility of cancer development in GC patients with the CSC markers overexpression. To assess the value of CSC markers overexpression on the prognosis of GC patients, pooled HRs with 95% CI values of survival outcomes, including OS, DFS/RFS, and CSS/DSS, were calculated. HRs were derived from both multivariate and univariate statistical tests by favoring information from multivariate statistics if applicable. When the Kaplan–Meyer curve was presented without declaring HR, it was calculated by Kaplan-Meyer curve according to the method described by Parmar et al. [17]. In this regard, the software GetData Graph Digitizer (http://getdata-graph-digitizer.com/) was utilized to extract survival data from Kaplan-Meyer curves. A pooled HR larger than 1 reflected a poor prognosis in GC patients. The heterogeneity among the included studies was determined through the I^2^ statistics. Random and fixed-effects models were employed for pooling the data based on the heterogeneity of the included studies. In the presence of considerable heterogeneity (P < 0.05 and/or an I^2^ statistic > 50%), random-effect models were applied; Otherwise, the fixed-effects models were utilized. Afterward, subgroup analyses on the basis of the expression of individual CSC markers were also employed to examine the possible cause of heterogeneity. Thereafter, the possible publication bias was graphically evaluated through funnel plots and statistically through Egger’s test. All these statistical analyses were conducted using the software Comprehensive Meta-Analysis Version 2.2.064. A two-tailed P < 0.05 defined statistical significance.

## Results

### Baseline study characteristics

**Fig. 1:**
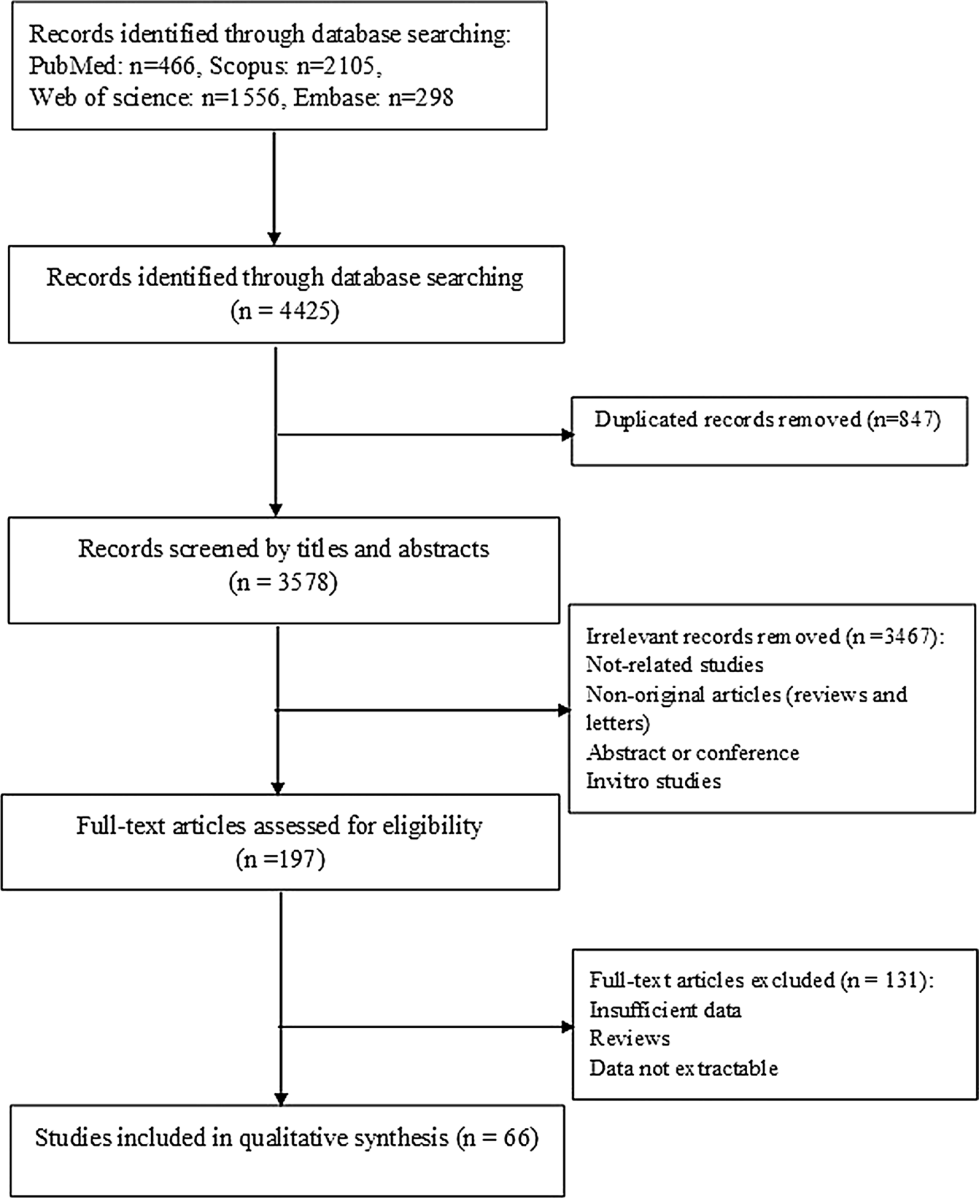
Flow chart of the literature search and selection procedure based on the preferred reporting items for systematic reviews and meta-analyses (PRISMA) guidelines

The details of the literature search and selection procedure are presented in a flowchart (Fig. 1). After carefully screening the titles, abstracts, and full text, a total of 66 publications, including 89 studies performed on 11,274 patients, were included for the present meta-analysis according to the inclusion criteria. Table 1 presents the main features of the included papers and patients’ demographics. Notably, all the eligible articles were written in English published between 2002 and 2020, with sample sizes ranging from 40 to 487 participants. According to the NOS quality assessment listed in Table 1, 66 publications were categorized as high quality ranged from 6 to 9. Geographically, most of the articles (n = 38) were carried out in China, while the remaining articles (n = 28) were conducted in other countries (Japan, Korea, Portugal, Netherlands, Germany, Turkey, Thailand, Egypt, Iran, Singapore, and Taiwan). Moreover, in the majority of studies, a large number of participants were male. All 66 articles applied the IHC detection method for analyzing tissue. Notably, 15 publications analyzed the same patient cohorts but by the use of different markers. To account for this purpose, each marker was incorporated in the related pooled analysis, while, for the total number of patients, these studies were only counted once. Among 66 relevant papers with 89 studies, several CSC markers (n = 13) were investigated; of them, 13 studies were performed on CD44s, 13 studies on CD133, 8 studies on Gli-1, 7 studies on Shh, 7 studies on Oct-4, 6 studies on Sox-2, 6 studies on Lgr-5, 6 studies on ALDH1, 5 studies on Bmi-1, 5 studies on CD44V6, 5 studies on CD44V9, 5 studies on CD24, and 3 studies on Nanog. The cut-off values were determined as a score on the basis of the intensity, percentage, or/and the number of positively stained cancer cells (Table 1). Furthermore, 78 studies evaluated the prognostic value of the CSC markers on OS, whilst 18 and 8 investigations assessed the prognostic importance of the markers on DFS/RFS and DSS/CSS, respectively.

**Table 1:** Main characteristics of the studies included in the meta-analysis

Authors year	CSC markers	Country duration	Mean/median age (month)	Sample size (n)	Gender (M/F)	TNM stage	Cut-off value	Follow-up (month)	Outcome	Hazard ratio (HR)	NOS score^#^
Wang 2011 [18]	CD44, CD133	Singapore 2000–2004	64	116	76/36	I–IV	CD44: 5 score (0–300) CD133: 50 score (0–300)	110	OS	E	8
Wakamatsu 2012 [19]	CD44, CD133, ALDH1	Japan NR	65	190	NR	I–IV	> 10%	83	OS	CD44, CD133: R-Multi ALDH1: R-Uni	6
Chen 2013 [20]	CD44, CD133	China 2000–2005	55 (23 to 84)	152	101/51	I–IV	CD44: > 65% CD133: > 50%	48.6 (1.0–76.7)	OS, DFS	OS: R-Multi DFS: E	9
Cao 2014 [21]	CD44	China 2005–2007	60.5 (33–88)	203	140/63	NR	> 10%	66 (7–108)	OS	E	6
Cao 2014 [22]	CD44, CD24	China 2000–2010	64 (32 to 87)	290	221/69	I–IV	≥ 30 (0–300)	41(3–135)	OS	R-Multi	9
Jian-Hui 2016 [23]	CD44, Gli-1, Shh	China 2006	60	101	62/39	I–III	≥ 4 score (0–12)	90	OS, DFS	CD44, Shh: R-Uni Gli: R-Multi	8
Zhang 2016 [24]	CD44, CD133, Sox-2, Oct-4, Gli-1	China 2005–2011	NR	101	NR	I–IV	> 10%	80	OS	E	6
Kodama 2017 [25]	CD44, CD44v6, CD44v9	Japan 2007–2009	70 (38–91)	123	83/40	I–IV	> 5%	68 (1–97)	DSS	R-Uni	7
Senel 2017 [26]	CD44, ALDH1	Turkey 2011–2015	NR	80	48/32	I–III	> 1 score (0–3)	60	DFS	E	7
Sun 2017 [27]	CD44	China 2004–2012	60	200	135/65	I–IV	> 3 score (0–6)	110	OS	E	8
Tongtawee 2017 [28]	CD44	Thailand 2011–2015	61.38 ± 12.39	162	117/45	I–IV	> 0 score (0–3)	60	OS	R-Multi	8
Ryu 2018 [29]	CD44	Korea 1998–2009	60.7(27–84)	143	91/52	NR	> 2 score (0–3)	45 (0–155)	OS, DFS	R-Multi	7
Ibrahim 2019 [30]	CD44	Egypt 2012–2016	52.5(41–60)	40	25/15	I–III	> 10%	8–36	OS, DFS	R-Uni	9
Xie 2015 [31]	CD44v6	China 2006–2008	60	208	154/54	I–IV	> 3 score	80	OS	R-Multi	8
Xu 2017 [32]	CD44v6	China 2006–2013	60	103	69/34	I–IV	NR	120	OS	E	7
Yamaguchi 2002 [33]	CD44v6	Japan 1984–1993	NR	201	NR	I–IV	NR	144	OS	E	6
Zheng 2017 [34]	CD44v6	China 2010–2015	65 (43–80)	49	39/10	NR	NR	33 (1–73)	DSS	R	9
Hirata 2013 [35]	CD44v9	Japan 2008–2010	67.5 (46–90)	65	56/9	NR	3.58 ± 7.74%	32 (1–36)	RFS	R-Multi	7
Yamakawa 2017 [36]	CD44v9	Japan 2011–2012	70	103	79/24	I–IV	≥ 4 score	61.0	RFS	R-Multi	8
Go 2016 [37]	CD44v9	*Korea* 1999–2007	24–85	333	218/115	I–III	> 0 score (0–3)	120	OS	E	8
Songun 2005 [38]	CD44v9	Netherlands 1989–1993	64.7 (31–84)	300	181 /119	I–IV	≥ 5%	120	OS	E	6
Ishigami 2010 [39]	CD133	*Japan* 2001–2003	65 (40 to 85)	97	69/28	I–IV	containing at least one CD133 positive cell	60	OS	E	8
Yu 2010 [40]	CD133	China 2004–2009	62.0 (29–83)	99	69/30	I–IV	NR	26.76 ± 17.02	OS	R-Multi	8
Zhao 2010 [41]	CD133	China NR	58.1 (18–85)	336	274/62	I–IV	≥ 5 score (0–12)	120	OS	E	7
Lee 2012 [42]	CD133	Korea 2001–2005	61.5 (29–89)	100	71/29	II–III	≥ 6 score (0–12)	46.9 (0–115)	OS, DFS	R-Multi	8
Hashimoto 2014 [43]	CD133	Japan 2004–2006	66 ± 11	189	133/56	I–IV	> 5%	60	OS	E	7
Zhou 2015 [44]	CD133, LGR-5	*China* 2004–2006	59.6 (23–88)	261	172/89	I–IV	> 2 score (0–12)	43.9 (0–110)	OS	R-Multi	7
Lu 2017 [45]	CD133	*China* 2010–2011	58.1 ( 28–78)	236	159/77	I–III	≥ 3 score (0–12)	48.6 (9–72)	OS	R-Multi	7
Attia 2019 [46]	CD133	Egypt 2012–2015	54.44 (24–81)	77	45/32	I–IV	> 3.5 score (0–6)	NR	RFS	R	6
Liu 2019 [47]	CD133, ALDH1	China 2012–2017	53 (32–76)	91	69/22	III	≥ 5 score (0–12)	27 (1–60)	OS, DFS	OS: E DFS:R-multi	7
Matsuoka 2012 [48]	Sox-2, Oct-4, Nanog	Japan NR	NR	290	NR	I–IV	≥ 5 score (0–5)	120	OS	R-Uni	6
Li 2015 [49]	SOX-2, Oct-4, Nanog	China 2008–2009	55 (28–78)	69	41/18	I–III	≥ 5 score (0–5)	35(6–60)	OS, DFS	E	8
Yang 2017 [50]	Sox-2, ALDH1	China 2010–2013	63 (29–82)	122	100/22	I–IV	> 5%	26 (1 to 75)	OS	Sox-2: R-Multi ALDH1: R-Uni	8
Zhang 2010 [51]	Sox-2	China 2004	57.8 ( 29–79)	50	35/15	I–IV	> 10%	1–50	OS	E	7
Camilo 2014 [52]	Sox-2	Portugal 1988–2010	66.5 (24–89)	201	124/77	I–IV	> 5%	250	OS	E	8
Kong 2014 [53]	Oct-4	China NR	60	158	104/54	I–IV	> 0 score (0–3)	60	OS	R-Multi	6
Jiang 2016 [54]	Oct-4	China 2001–2010	62 (30–85)	412	284/128	I–IV	≥ 4 score	60	OS	R-Multi	7
Javanbakht 2017 [55]	Oct-4	Iran 2010–2014	59.3 (37–85)	40	16/24	I–IV	> 25%	50	OS	R-Multi	6
El-Guindy 2019 [56]	Oct-4	Egypt 2015–2016	18–70	45	28/17	I–III	≥ 6 score (0–9)	24	OS, DFS	E	6
Li 2014 [57]	ALDH1	China 2005–2008	57.0 ( 22–82)	216	140/76	I–III	≥ 2 score (0–3)	27 (4–82)	OS, RFS	R-Multi	8
41Lu 2018 [58]	ALDH1	*China* 2011–2012	60	232	148/84	I–III	> 2 score (0–12)	48.7 (10–83)	OS	R-Multi	7
Simon 2012 [59]	LGR-5	Germany 1997–2009	68 + _11.4	487	304/183	I–IV	NR	60.9 (14.3–129.9)	OS	E	6
Bu 2013 [60]	LGR-5	*China* 2002–2007	61 (22–87)	257	185/72	I–IV	≥ 3 score (0–6)	60	OS	R-Uni	8
Xi 2014 [61]	LGR-5	China 1999–2004	59.6 (24–86)	318	254/59	I–IV	≥ 2 score (0–6)	120	OS	R-Multi	7
Choi 2017 [62]	LGR-5	Korea 2004–2006	60	456	312/144	I–IV	NR	NR	CSS	R-Multi	6
Liu 2019 [63]	LGR-5	*China* 2009–2014	60 (33 to 85)	100	68/32	I–III	> 6 score (0–12)	60	OS	E	8
Liu 2008 [64]	Bmi-1	China 1999–2002	60	146	92/54	I–IV	≥ 10%	48	OS	R-Multi	7
Zhang 2010 [65]	Bmi-1	China NR	60	75	50/25	I–IV	≥ 10%	110	OS	E	7
Yang 2011 [66]	Bmi-1	China 2004–2005	60	219	162/57	I–IV	≥ 20%	29.57 (1.90–78.10)	DSS	E	7
Wu 2016 [67]	Bmi-1	China NR	60	352	236/116	I–IV	NR	120	OS	E	6
Lu 2012 [68]	Bmi-1	China 1993–2006	55	309	98/211	NR	> 5%	67.8 (2–200)	OS	E	7
Lin 2012 [69]	Nanog	China NR	50	105	71/34	I–IV	> 2 score (0–12)	60	OS	R-Multi	7
Chou 2007 [70]	CD24	Taiwan 1995–1997	73 (40–99)	103	75/28	I–IV	> 10%	86.5 (1–120)	OS	R-Multi	6
Fujikuni 2014 [71]	CD24	Japan 2001–2008	65	119	73/46	I–III	> 1%	60	OS	E	6
Darwish 2004 [72]	CD24	Korea NR	54.3	300	200/100	I–IV	NR	53 (1–72)	OS	R-Multi	8
Bektas 2010 [73]	CD24	Turkey 2001–2009	61.5 (30–84)	93	59/34	I–IV	> 10%	36 (0–100)	OS	E	8
Hu 2017 [74]	Gli-1, Shh	China NR	60	90	53/37	I–IV	≥ 6 score (0–12)	80	OS	E	6
Shao 2017 [75]	Gli-1	China 2008–2010	60	67	50/17	I–IV	≥ 5 score (0–12)	60	OS	R-Multi	9
*Tang* 2018 [76]	Gli-1	China 2009–2010	60	90	68/22	I–IV	> 3 score (0–9)	80	OS	R-Multi	7
Ke 2020 [77]	Gli-1, Shh	China 2010–2013	60	128	128/50	I–III	> 3 score	120	OS	R-Multi	8
Wang 2014 [78]	Gli-1	China 2005–2007	63 (46–83)	121	92/29	I–IV	≥ 10%	30 ( 5–60)	OS	R-Multi	7
Yao 2019 [79]	Gli-1	China 2010–2012	60	57	40/17	I–IV	≥ 5 score (0–12)	60	OS	E	9
Ertao 2016 [80]	Shh	China 2004–2005	60	117	71/46	I–IV	> 10%	47.6 (3–114)	OS	R-Multi	8
Kim 2012 [81]	Shh	Korea 2004–2007	NR	319	NR	I–IV	> 3 score (0–4)	80	OS	E	6
Yoo 2011 [82]	Shh	Korea 2002–2004	60	178	124/49	I–IV	NR	60	OS	E	7
Niu 2014 [83]	Shh	China 2008–2009	54.37 (22–75)	113	73/40	I–IV	≥ 2 score (0–3)	43.6 (6–78)	OS	R-Multi	7

HR: Hazard ratio; OS: Overall survival; RFS: Relapse-free survival; DSS: Disease-specific survival; DFS: Disease-free survival; CSS: Cancer-specific survival; NR: Not reported; IHC: Immunohistochemistry; E: Estimated by survival curve; R: Reported; R-Uni: Univariate analysis reported by article; R-Multi: Multivariate analysis reported by article; # Study quality was evaluated according to the Newcastle–Ottawa Scale (range, 1–9)

### Association between cancer stem cell (CSC) markers expression and clinicopathological features

Table 2 exhibits findings of the relationship between CSC markers expression and the clinicopathological parameters. Overall analyses of the markers indicated that overexpression of CSC markers is significantly associated with TNM stage (OR = 2.19, 95% CI 1.84–2.61, *P* = 0.013) and lymph node metastasis (OR = 1.76, 95% CI 1.54–2.02, *P* < 0.001) with considerable heterogeneity (I^2^ = 80.74%, *P*_*h*_ < 0.001 and I^2^ = 73.57%, *P*_*h*_ < 0.001), respectively. No significant association was found between CSC markers and tumor differentiation (OR = 1.25, 95% CI 1.01–1.55, *P* = 0.035; I^2^ = 82.38%, *P*_*h*_ < 0.001). Thereafter, we assessed the association between the expression of individual CSC markers and clinicopathological features. As shown in Table 2, the expressions of ALDH1 (OR = 3.66, 95% CI 1.75–7.64, *P* < 0.001; I^2^ = 74.19%, *P*_*h*_ = 0.009), Bmi-1 (OR = 2.85, 95% CI 1.04–7.81, *P* = 0.041; I^2^ = 88.46%, *P*_*h*_ < 0.001), CD133 (OR = 2.67, 95% CI 1.84–3.89, *P* < 0.001; I^2^ = 54.01%, *P*_*h*_ = 0.016), CD44 (OR = 2.74, 95% CI 1.39–5.38, *P* = 0.003; I^2^ = 83.96%, *P*_*h*_ < 0.001), CD44V6 (OR = 2.50, 95% CI 1.22–5.14, *P* = 0.012; I^2^ = 70.46%, *P*_*h*_ = 0.034), CD44V9 (OR = 5.39, 95% CI 2.70–10.77, *P* < 0.001; I^2^ = 7.26%, *P*_*h*_ = 0.29), Gli-1 (OR = 4.00, 95% CI 1.58–10.13, *P* = 0.003; I^2^ = 79.50%, *P*_*h*_ < 0.001), and Oct-4 (OR = 2.25, 95% CI 1.09–4.66, *P* = 0.028; I^2^ = 81.91%, *P*_*h*_ < 0.001) were associated with TNM stage. Moreover, it was found that only CD44s expression is statistically linked to tumor differentiation (OR = 2.82, 95% CI 1.50–5.30, *P* = 0.001; I^2^ = 75.38%, *P*_*h*_ < 0.001). Additionally, there was a relationship between the expressions of Oct-4 (OR = 2.70, 95% CI 1.09–6.68, *P* = 0.031; I^2^ = 88.63%, *P*_*h*_ < 0.001), Bmi-1 (OR = 2.26, 95% CI 1.19–4.26, *P* = 0.012; I^2^ = 76.82%, *P*_*h*_ = 0.002), CD133 (OR = 1.85, 95% CI 1.22–2.79, *P* = 0.003; I^2^ = 65.05%, *P*_*h*_ = 0.001), CD44 (OR = 1.64, 95% CI 1.13–2.38, *P* = 0.009; I^2^ = 56.83%, *P*_*h*_ = 0.01), CD44V6 (OR = 2.26, 95% CI 1.46–3.51, *P* < 0.001; I^2^ = 25.64%, *P*_*h*_ = 0.25), CD24 (OR = 1.40, 95% CI 1.04–1.89, *P* = 0.026; I^2^ = 20.17%, *P*_*h*_ = 0.28), Gli-1 (OR = 3.04, 95% CI 1.62–5.71, *P* = 0.001; I^2^ = 51.59%, *P*_*h*_ = 0.08), and Sox-2 (OR = 1.96, 95% CI 1.40–2.73, *P* < 0.001; I^2^ = 0.00%, *P*_*h*_ = 0.42) and lymph node metastasis. However, no clear association was identified between some CSC markers, including LGR-5, Nanog, or Shh, and the clinicopathological features (all; *P* > 0.05).

**Table 2:** Main results of pooled HRs in the meta-analysis

	Study number	Sample size	OR/HR (95% CI)	P value	z	Heterogeneity		Publication bias (Eggers test)	
						I^2^ (%)	P_h_	T-value	P_bias_
Overall TNM stage (III/IV vs I/II)	70	11,901	2.19 (1.84–2.61)	P < 0.001	8.83	80.74	*P* < 0.001	2.40	0.018
Overall tumor differentiation (poor vs well/moderate)	59	9251	1.25 (1.01–1.55)	0.035	2.10	82.38	*P* < 0.001	1.84	0.07
Overall lymph node metastasis (Yes vs No)	76	13,172	1.76 (1.54–2.02)	0.00	8.23	73.57	*P* < 0.001	2.37	0.019
Overall OS	78	13,482	1.65 (1.54–1.77)	0.00	14.00	56.74	*P* < 0.001	3.70	*P* < 0.001
Overall DFS/RFS	18	1788	2.35 (1.90–2.89)	P < 0.001	7.96	46.93	0.015	1.63	0.12
Overall CSS/ DSS	8	1462	1.69 (1.33–2.15)	P < 0.001	4.32	49.04	0.056	1.00	0.35
CD44s									
TNM stage (III/IV vs I/II)	9	1331	2.74 (1.39–5.38)	0.003	2.93	83.96	*P* < 0.001	1.55	0.163
Tumor differentiation (poor vs well/moderate)	9	1177	2.82 (1.50–5.30)	0.001	3.23	75.38	*P* < 0.001	0.71	0.49
Lymph node metastasis (Yes vs No)	11	1638	1.64 (1.13–2.38)	0.009	2.62	56.83	0.010	1.65	0.13
OS	11	1669	1.97 (1.55–2.50)	P < 0.001	5.61	54.99	0.014	1.62	0.13
DFS/RFS	5	516	2.74 (1.80–4.17)	P < 0.001	4.69	42.23	0.14	–	–
CSS/DSS	2	246	2.59 (1.32–5.06)	0.005	2.78	0.00	0.65	–	–
CD44V6									
TNM stage (III/IV vs I/II)	3	311	2.50 (1.22–5.14)	0.012	2.51	70.46	0.034	0.40	0.75
Tumor differentiation (poor vs well/moderate)	5	684	0.93 (0.35–2.42)	0.88	− 0.14	86.80	*P* < 0.001	0.55	0.61
Lymph node metastasis (Yes vs No)	5	684	2.26 (1.46–3.51)	P < 0.001	3.65	25.64	0.25	1.68	0.19
OS	3	512	1.81 (1.29–2.53)	0.001	3.45	0.00	0.82	2.34	0.25
CSS/DSS	3	295	3.29 (1.46–7.43)	0.004	2.87	61.63	0.10	–	–
CD44V9									
TNM stage (III/IV vs I/II)	2	637	5.39 (2.70–10.77)	P < 0.001	4.78	7.26	0.29	–	–
Tumor differentiation (poor vs well/moderate)	3	559	0.98 (0.37–2.58)	0.98	− 0.025	84.72	0.001	14.65	0.043
Lymph node metastasis (Yes vs No)	3	559	1.52 (0.91–2.52)	0.10	1.61	40.74	0.18	8.64	0.073
OS	2	619	1.22 (0.96–1.54)	0.08	1.69	*P* < 0.001	0.74	–	–
DFS/RFS	2	168	8.60 (1.70–43.57)	0.009	2.61	77.51	0.035	–	–
CSS/DSS	2	246	1.67 (0.52–5.35)	0.38	0.86	0.00	0.48	–	–
CD133									
TNM stage (III/IV vs I/II)	11	1853	2.67 (1.84–3.89)	P < 0.001	5.16	54.01	0.016	0.25	0.80
Tumor differentiation (poor vs well/moderate)	10	1677	0.87 (0.56–1.34)	0.53	− 0.62	70.18	*P* < 0.001	2.09	0.06
Lymph node metastasis (Yes vs No)	11	1847	1.85 (1.22–2.79)	0.003	2.93	65.05	0.001	0.99	0.34
OS	12	1939	1.74 (1.52–2.01)	*P* < 0.001	7.87	0.00	0.55	1.83	0.09
DFS/RFS	3	343	2.59 (1.74–3.85)	*P* < 0.001	4.69	0.00	0.64	–	–
Sox-2									
TNM stage (III/IV vs I/II)	5	732	1.37 (0.65–2.90)	0.40	0.83	77.22	0.002	1.55	0.21
Tumor differentiation (poor vs well/moderate)	2	119	1.21 (0.57–2.59)	0.60	0.51	23.37	0.25	–	–
Lymph node metastasis (Yes vs No)	5	732	1.96 (1.40–2.73)	*P* < 0.001	3.99	0.00	0.42	0.84	0.46
OS	6	804	1.73 (1.37–2.18)	*P* < 0.001	4.63	0.00	0.78	0.59	0.58
DFS/RFS	1	69	1.31 (0.68–2.49)	0.41	0.82	0.00	1	–	–
Oct-4									
TNM stage (III/IV vs I/II)	6	1014	2.25 (1.09–4.66)	0.028	2.19	81.91	*P* < 0.001	0.79	0.47
Tumor differentiation (poor vs well/moderate)	5	724	1.97 (0.86–4.50)	0.10	1.60	75.47	0.003	0.53	0.62
Lymph node metastasis (Yes vs No)	6	1014	2.70 (1.09–6.68)	0.031	2.15	88.63	*P* < 0.001	1.15	0.31
OS	7	1086	1.87 (1.48–2.35)	0.00	5.37	4.88	0.38	0.21	0.83
DFS/RFS	2	114	2.18 (1.06–4.48)	0.023	2.13	11.13	0.28	–	–
ALDH1									
TNM stage (III/IV vs I/II)	4	760	3.66 (1.75–7.64)	0.001	3.45	74.19	0.009	1.31	0.31
Tumor differentiation (poor vs well/moderate)	3	539	2.73 (0.43–17.19)	0.28	1.07	93.25	*P* < 0.001	4.6	0.13
Lymph node metastasis (Yes vs No)	4	760	2.50 (0.96–6.45)	0.058	1.89	88.08	*P* < 0.001	0.09	0.93
OS	5	851	1.65 (1.32–2.05)	*P* < 0.001	4.53	0.00	0.49	2.60	0.08
DFS/RFS	2	307	1.49 (0.73–3.00)	0.27	1.10	56.39	0.13	–	–
LGR-5									
TNM stage (III/IV vs I/II)	5	1392	1.31 (0.46–3.74)	0.60	0.51	94.11	*P* < 0.001	0.58	0.6
Tumor differentiation (poor vs well/moderate)	5	1423	1.04 (0.50–2.13)	0.91	0.10	87.79	*P* < 0.001	0.36	0.73
Lymph node metastasis (Yes vs No)	6	1879	1.21 (0.70–2.09)	0.48	0.70	84.70	*P* < 0.001	0.01	0.98
OS	5	1879	1.26 (0.97–1.63)	0.075	1.78	60.45	0.039	1.03	0.37
CSS/DSS	1	456	1.02 (0.67–1.54)	0.90	0.11	0.00	1.00	–	–
Bmi-1									
TNM stage (III/IV vs I/II)	4	792	2.85 (1.04–7.81)	0.041	2.04	88.46	*P* < 0.001	1.07	0.36
Tumor differentiation (poor vs well/moderate)	4	792	1.14 (0.70–1.85)	0.57	0.55	51.72	0.10	0.09	0.93
Lymph node metastasis (Yes vs No)	5	1101	2.26 (1.19–4.26)	0.012	2.51	76.82	0.002	1.13	0.33
OS	4	882	1.32 (0.77–2.27)	0.30	1.06	84.49	*P* < 0.001	1.57	0.25
CSS/DSS	1	219	1.97 (1.35–2.87)	*P* < 0.001	3.52	0.00	1.00	–	–
Nanog									
TNM stage (III/IV vs I/II)	3	464	1.34 (0.78–2.31)	0.27	1.08	0.86	*P* < 0.001	0.49	0.70
Tumor differentiation (poor vs well/moderate)	2	174	1.89 (0.06–55.82)	0.71	0.37	93.05	*P* < 0.001	–	–
Lymph node metastasis (Yes vs No)	3	464	1.49 (0.73–3.02)	0.26	1.10	44.43	0.16	0.71	0.60
OS	3	464	1.59 (0.68–3.77)	0.28	1.07	80.97	0.005	0.93	0.52
DFS/RFS	1	69	2.09 (0.92–4.70)	0.075	1.77	0.00	1	–	–
CD24									
TNM stage (III/IV vs I/II)	5	905	1.31 (0.87–1.98)	0.18	1.32	43.46	0.13	0.91	0.42
Tumor differentiation (poor vs well/moderate)	2	393	1.10 (0.18–6.46)	0.91	0.10	90.02	0.002	–	–
Lymph node metastasis (Yes vs No)	5	905	1.40 (1.04–1.89)	0.026	2.23	20.17	0.28	0.46	0.67
OS	5	905	1.73 (1.25–2.4)	0.001	4.10	35.46	0.18	2.71	0.07
Gli-1									
TNM stage (III/IV vs I/II)	6	614	4.00 (1.58–10.13)	0.003	2.93	79.50	*P* < 0.001	1.07	0.34
Tumor differentiation (poor vs well/moderate)	4	403	1.66 (0.81–3.40)	0.16	1.39	57.08	0.07	0.93	0.44
Lymph node metastasis (Yes vs No)	5	493	3.04 (1.62–5.71)	0.001	3.47	51.59	0.08	0.60	0.59
OS	8	776	1.75 (1.34–2.31)	0.00	4.06	7.79	0.37	0.14	0.88
DFS/RFS	1	101	3.40 (1.30–8.7)	0.012	2.52	0.00	1	–	–
Shh									
TNM stage (III/IV vs I/II)	7	1096	2.19 (0.97–4.94)	0.056	1.90	86.32	*P* < 0.001	3.12	0.026
Tumor differentiation (poor vs well/moderate)	5	687	1.02 (0.24–4.30)	0.97	0.037	90.31	*P* < 0.001	1.63	0.20
Lymph node metastasis (Yes vs No)	7	1096	1.14 (0.54–2.39)	0.73	0.34	85.02	*P* < 0.001	0.48	0.64
OS	7	1096	1.27 (0.78–2.05)	0.32	0.99	76.23	*P* < 0.001	0.72	0.49
DFS/RFS	1	101	2.75 (1.15–6.54)	0.02	2.28	0.00	1	–	–

P_h_: The p-value of heterogeneity; P _bias_: The p-value of Egger test for assessing publication bias; OS: Overall survival; DFS: Disease-free survival; RFS: Relapse-free survival; CSS: Cancer-specific survival; DSS: Disease-specific survival

### Association between cancer stem cell (CSC) markers expression and overall survival (OS)

**Fig. 2:**
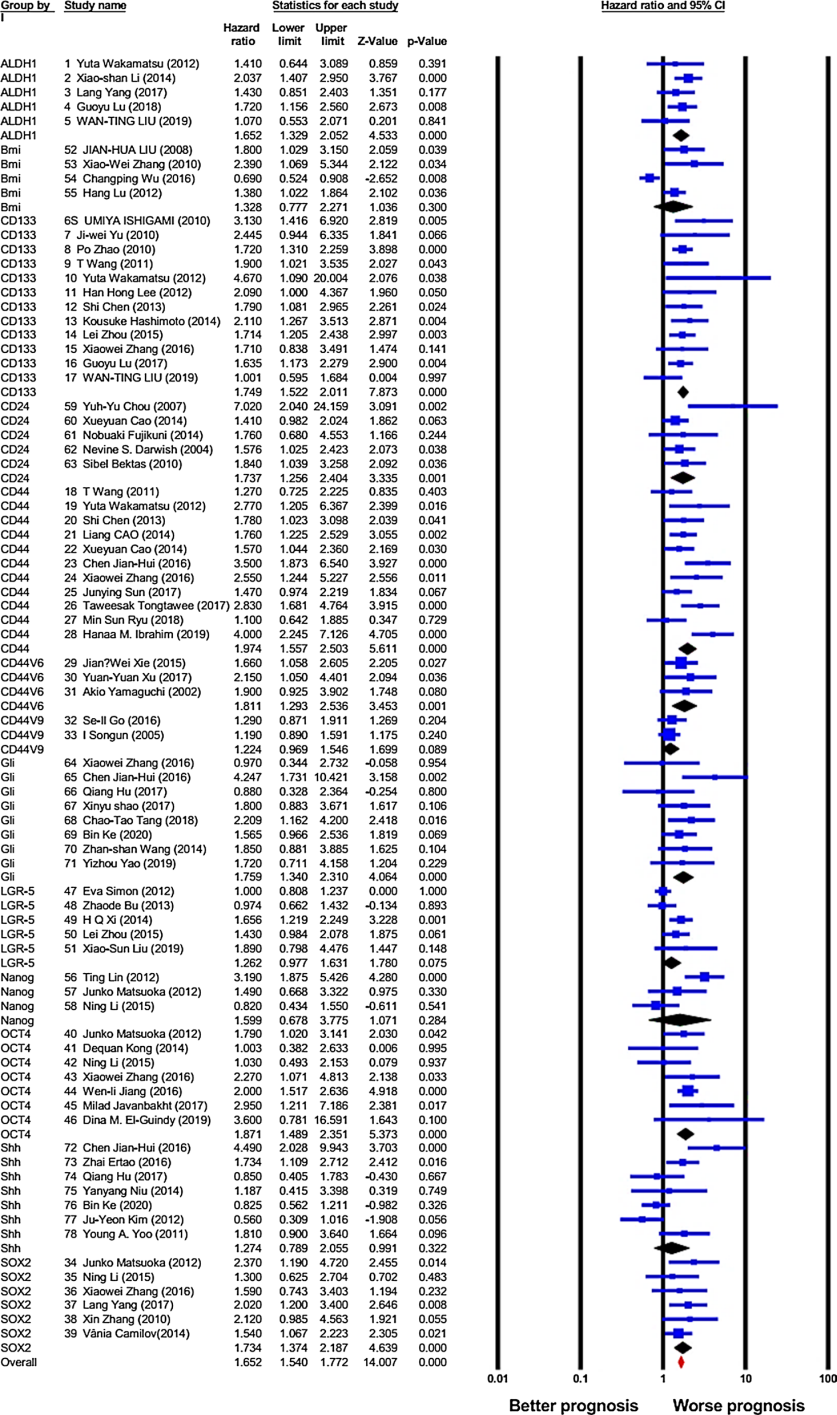
Forest plot showing the results of the association between cancer stem cell (CSC) markers expression and overall survival (OS) in gastric cancer (GC)

A total of 78 studies provided adequate information to evaluate the link between CSC markers and OS in patients with GC, and as presented in Fig. 2 and Table 2, overexpression of overall CSC markers notably predicted worse OS in GC patients (HR = 1.65, 95% CI 1.54–1.77, *P* < 0.001), with a rather moderate heterogeneity (I^2^ = 56.74%, *P* < 0.001). Consequently, a random-effect model was utilized. Afterward, the relationship between expression of individual CSC markers and OS was studied. Accordingly, the obtained results showed that overexpression of ALDH1 (HR = 1.65, 95% CI 1.32–2.05, *P* < 0.001; I^2^ = 0.00%, *P*_*h*_ = 0.49), CD133 (HR = 1.74, 95% CI 1.52–2.01, *P* < 0.001; I^2^ = 0.00%, *P*_*h*_ = 0.55), CD24 (HR = 1.73, 95% CI 1.25–2.4, *P* < 0.001; I^2^ = 33.46%, *P*_*h*_ = 0.18), CD44 (HR = 1.97, 95% CI 1.55–2.50, *P* < 0.001; I^2^ = 54.99%, *P*_*h*_ = 0.014), CD44V6 (HR = 1.81, 95% CI 1.29–2.53, *P* = 0.001; I^2^ = 0.00%, P_*h*_ = 0.82), Gli-1 (HR = 1.75, 95% CI 1.34–2.31, *P* < 0.001; I^2^ = 7.79%, *P*_*h*_ = 0.37), Oct-4 (HR = 1.87, 95% CI 1.48–2.35, *P* < 0.001; I^2^ = 4.88%, *P*_*h*_ = 0.38), and Sox-2 (HR = 1.73, 95% CI 1.37–2.18, *P* < 0.001; I^2^ = 0.00%, *P*_*h*_ = 0.78) are positively linked with worse OS in GC patients. However, data analysis indicated no statistically meaningful association between the overexpression of Bmi-1 (HR = 1.32, 95% CI 0.77–2.27, *P* = 0.3; I^2^ = 84.49%, *P*_*h*_ < 0.001), CD44V9 (HR = 1.22, 95% CI 0.96–1.54, *P* = 0.08; I^2^ = 0.00%, *P*_*h*_ = 0.74), LGR-5 (HR = 1.26, 95% CI 0.97–1.63, *P* = 0.07; I^2^ = 60.45%, *P*_*h*_ = 0.039), Nanog (HR = 1.59, 95% CI 0.67–3.77, *P* = 0.28; I^2^ = 80.97%, *P*_*h*_ = 0.005) or Shh (HR = 1.27, 95% CI 0.78–2.05, *P* = 0.32; I^2^ = 76.23%, *P*_*h*_ < 0.001) and OS.

### Association between cancer stem cell (CSC) markers expression and disease-free survival (DFS)/relapse-free survival (RFS)

**Fig. 3:**
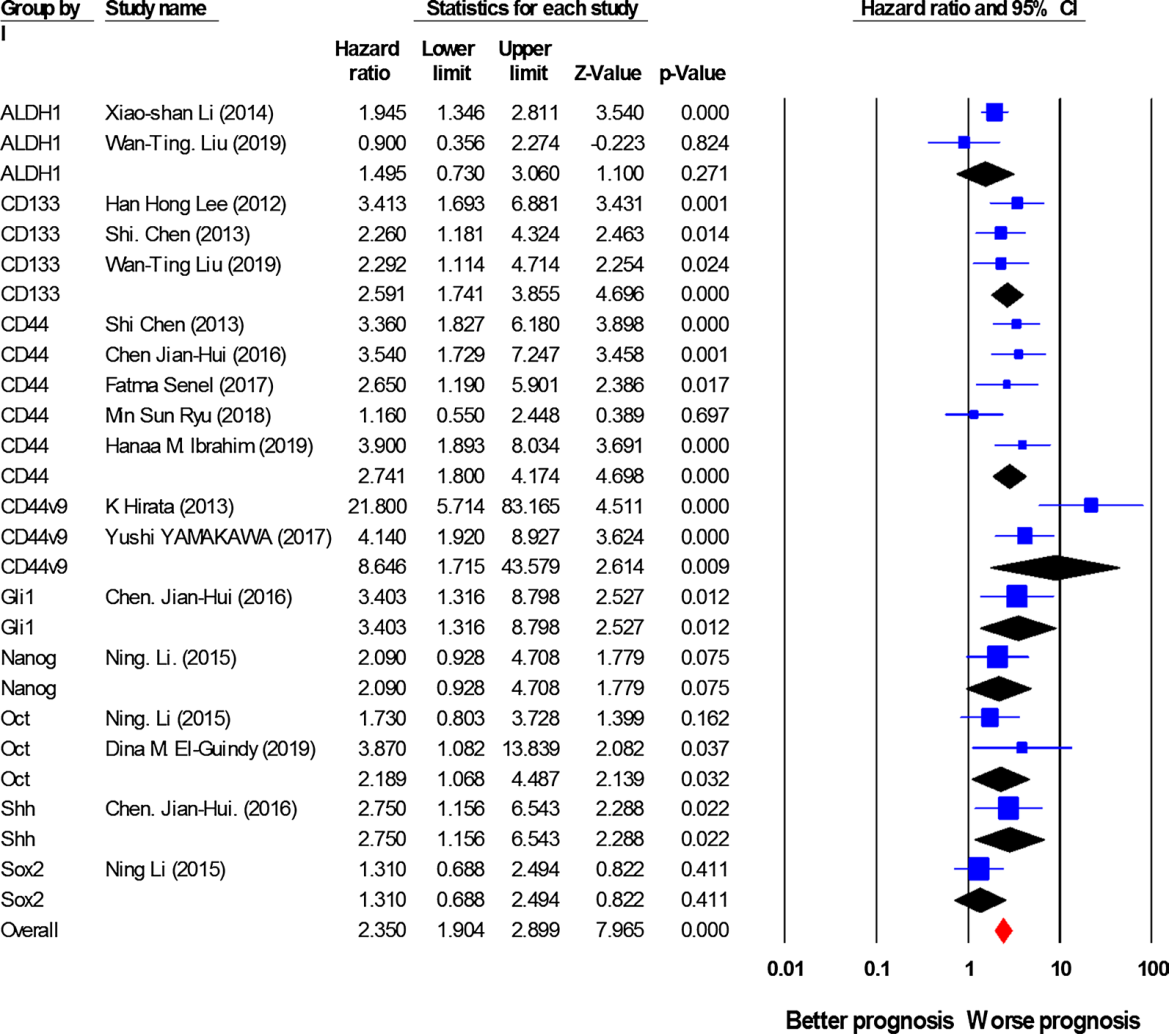
Forest plot showing the results of the association between cancer stem cell (CSC) markers expression and disease-free survival/ relapse-free survival (DFS/RFS) in gastric cancer (GC)

18 studies including 1788 patients investigated the relationship between expression of CSC markers and DFS/RFS. As shown in Fig. 3 and Table 2, the overall pooled HR indicated a considerable elevated risk of disease progression or recurrence in the cases with high expression of CSC markers (HR = 2.35, 95% CI 1.90–2.89, *P* < 0.001), with a rather slight heterogeneity (I^2^ = 46.93%, *P* = 0.015). Analyses for individual CSC markers indicated that high expression of CSC markers, including CD133 (HR = 2.59, 95% CI 1.74–3.85, *P* < 0.001; I^2^ = 0.00%, *P*_*h*_ = 0.64), CD44 (HR = 2.74, 95% CI 1.80–4.17, *P* < 0.001; I^2^ = 42.23%, *P*_*h*_ = 0.14), CD44V9 (HR = 8.60, 95% CI 1.70–43.57, *P* = 0.009; I^2^ = 77.51%, *P*_*h*_ = 0.035), and Oct-4 (HR = 2.18, 95% CI 1.06–4.48, *P* = 0.023; I^2^ = 4.88%, *P*_*h*_ = 0.38) is associated with a poor DFS/RFS. Additionally, a limited number of publications reported the association of Gli-1 (n = 1, HR = 3.40, 95% CI 1.30–8.70, *P* = 0.012) and Shh (n = 1, HR = 2.75, 95% CI 1.15–6.54, *P* = 0.02) with DFS/RFS. However, data analysis showed no remarkable effects of overexpression of ALDH1 (HR = 1.49, 95% CI 0.73–3, *P* = 0.27; I^2^ = 0.00%, *P*_*h*_ = 0.49), Nanog (HR = 2.09, 95% CI 0.92–4.7, *P* = 0.075; I^2^ = 80.97%, *P*_*h*_ = 0.005) or Sox-2 (HR = 1.31, 95% CI 0.68–2.49, *P* = 0.41; I^2^ = 0.00%, *P*_*h*_ = 0.78) on DFS/RFS. Furthermore, in this study, the relationships between overexpression of Bmi-1, CD24, CD44v6 or LGR-5 and DFS/RFS were not investigated due to the lack of sufficient information.

### Association between cancer stem cell (CSC) markers expression and cancer-specific survival (CSS)/ Disease-specific survival (DSS)

**Fig. 4:**
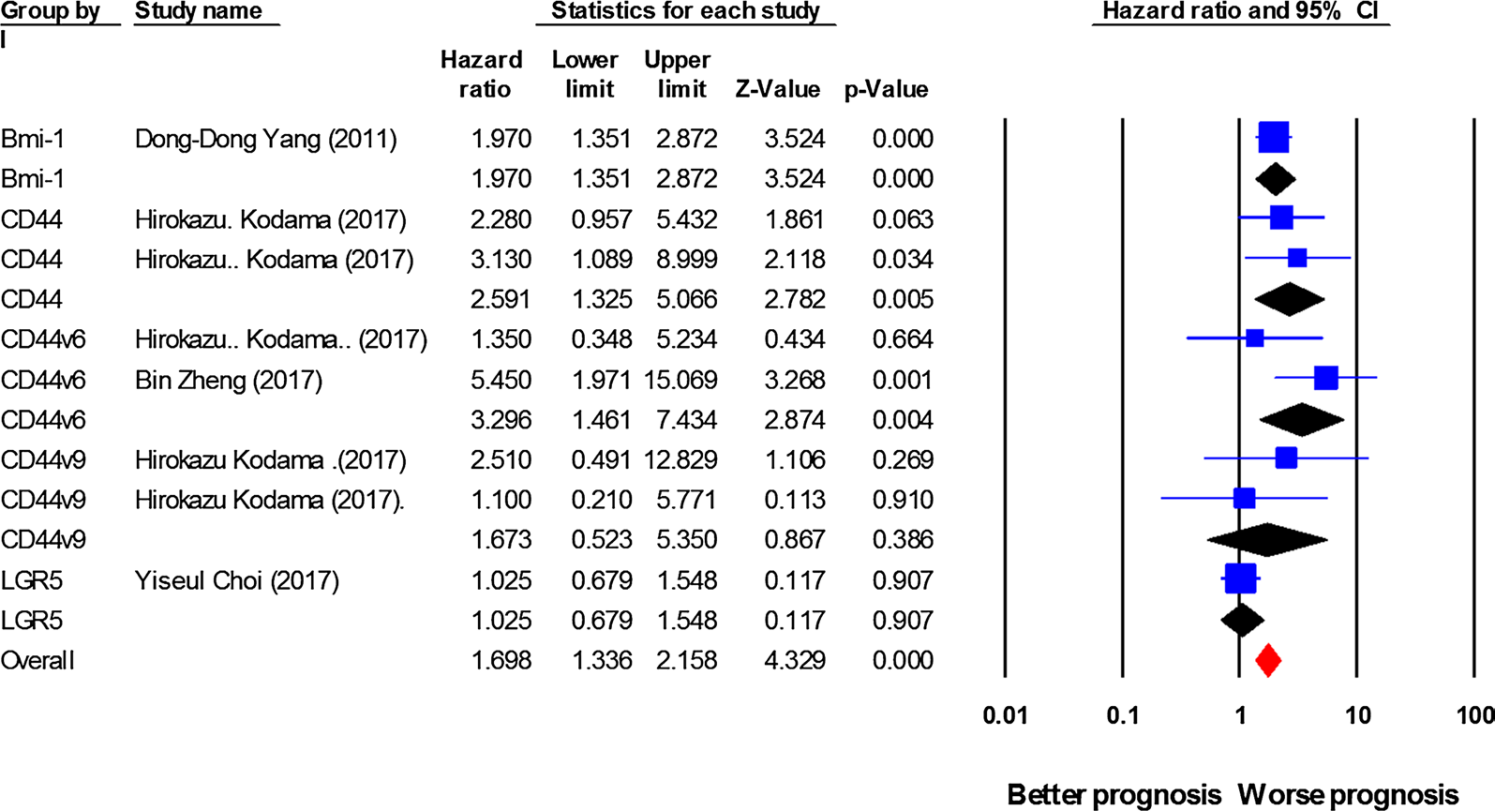
Forest plot showing the results of the association between cancer stem cell (CSC) markers expression and cancer-specific survival/ Disease-specific survival (CSS/ DSS) in gastric cancer (GC)

HRs for CSC markers were presented in 8 studies, involving 1462 cases. The pooled HR indicated a considerable prognostic importance of CSC markers overexpression in GC patients CSS/DSS prediction (HR = 1.69, 95% CI 1.33–2.15, *P* < 0.001, Fig. 4 and Table 2), with a slight heterogeneity (I^2^ = 49.04%, *P* = 0.056). From one study, more than one HR was extracted for CSS/DSS, because the expression of multiple CSC markers was investigated [25]. A subgroup analysis on the basis of the expression of CSC markers indicated that although Bmi-1 (HR = 1.97, 95% CI 1.35–2.87, *P* < 0.001), CD44s (HR = 2.59, 95% CI 1.32–5.06, *P* = 0.005; I^2^ = 61.63%, *P*_*h*_ = 0.1), and CD44V6 (HR = 3.29, 95% CI 1.46–7.43, *P* = 0.004; I^2^ = 61.63%, *P*_*h*_ = 0.1) are significantly associated with GC patients CSS/DSS, CD44V9 (HR = 1.67, 95% CI 0.52–5.35, *P* = 0.38; I^2^ = 0.00%, *P*_*h*_ = 0.48) and LGR-5 (HR = 1.02, 95% CI 0.67–1.54, *P* = 0.9) are not associated. Moreover, the associations between ALDH1, CD133, CD24, Oct-4, Gli-1, Shh, Sox-2, Nanog, or LGR-5 expression and CSS/DSS were not studied owing to the inadequacy of data.

### Publication Bias

**Fig. 5:**
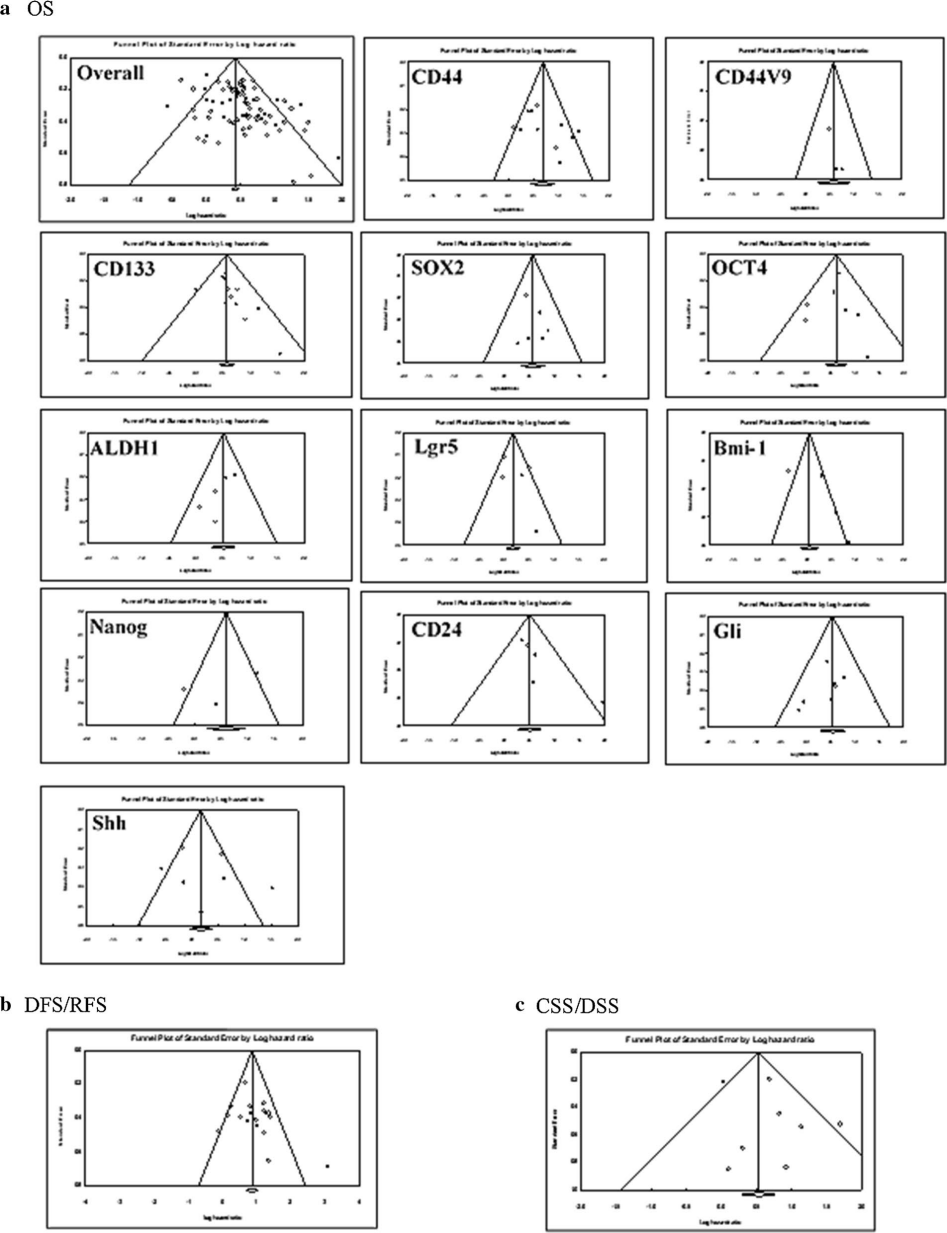
Funnel plots for publication bias test with 95%confidence limits. **a** Cancer stem cell (CSC) markers associated with overall survival (OS); **b** CSC markers associated with disease-free survival/ relapse-free survival (DFS/RFS); **c** CSC markers associated with cancer-specific survival/ Disease-specific survival (CSS/DSS)

In the current meta-analysis, the presence of the publication bias in the eligible studies was evaluated for OS, DFS/RFS, and DSS/CSS using funnel plot analysis (Fig. 5) and Egger’s test (Table 2). Graphically, the funnel plots showed that asymmetry might be present in the graph of the studies concerning overall OS, but probably not in DFS/RFS and DSS/CSS studies (Fig. 5), proposing the possible existence of a publication bias about OS. Subsequently, Egger’s tests were conducted to investigate the bias more precisely. The studies regarding the expression of overall CSC markers and OS (Table 2) demonstrated a significant publication bias as evaluated through Egger’s test (t-value = 3.7, *P* < 0.001). Then, the publication bias was evaluated for individual CSC markers and OS, in which Egger’s test and funnel plot graphs revealed nonsignificant publication bias for each CSC marker. However, we did not perform the publication bias analysis for the relationship between the expression of individual CSCs markers and DFS/RFS or DSS/CSS because of the limited number of eligible studies.

## Discussion

This review provided the most comprehensive meta-analysis of gastric CSC biomarkers and recognized clinicopathological and prognostic significances for CSC markers. Our meta-analysis presented robust evidence for the association between CSC biomarkers expression and GC prognosis by enrolling 11,274 GC participants, emphasizing the potential clinical applicability of CSC biomarkers in GC. Principally, we attempted to address the study heterogeneity and publication bias.

Generally, we identified a strong association between higher levels of CSC biomarkers and TNM stage, lymph node metastasis, poor OS, DFS/RFS, and CSS/DSS, suggesting their important roles in prognosis and patient stratification. These findings suggest that gastric CSC markers may assist clinicians and decision-makers in evaluating GC status after surgery. However, the overall expression of CSC markers had a slight association with tumor differentiation of GC cells. Based on the obtained findings, CSC markers are likely to have a more key role in the relapse of GC (HR = 2.35) compared to death resulted from GC (HR = 1.69). However, because of the limited sample sizes regarding CSS/DSS and DFS/RFS, our findings should be interpreted with caution. Data involving clinical value and prognostic significance of overall CSC markers expression were characterized by partially high heterogeneity, and, to specify the positive staining for each marker, a considerable variability within the cut-off thresholds utilized in the various studies was identified. Variability of patients’ characteristics and the different antibodies used for the detection of CSC markers might lead to heterogeneity among these investigations. Additionally, publication bias was detected in the pooled HR for the OS and overall CSC markers expression. However, analyses based on the individual expression of CSC markers were free of any publication bias.

Gastric CSC markers have been suggested to interact with each other and with several signaling pathways, which were found to be associated with aggressive phenotype [84, 85]. Accumulating evidence has displayed that CSCs can promote growth, migration, angiogenesis, and metastasis of gastric tumor cells, which may support the association between the expression of CSC markers and clinical outcomes [86, 87]. CSCs are a minor subpopulation within the total cancer cells, making their identification in the heterogeneous masses of cells challenging. The appropriate approach to detect and target CSCs has been determined to be the utilization of cell-surface markers in different cancer types [88]. However, a lack of uniform expression of the already used markers might limit their advantages for CSCs detection, and, notably, inconsistencies still remain regarding the ideal markers panel to detect gastric CSCs. CD44, a transmembrane glycoprotein, is the first identified cell-surface marker used commonly for the isolation of CSCs [89], and subsequently, the associations of CD24, CD44v6, CD44v9, CD133, LGR-5, and cytosolic ALDH1 with the clinicopathological parameters of cancers have been investigated in various studies [19, 90–92]. In GC patient-derived xenografts, tumor biopsies, and cell lines, the fraction of cells with overexpression of these CSC markers displays self-renewal, tumorigenic, multilineage differentiation, and chemoresistance abilities, proposing that these may be robust CSC biomarkers [12, 93]. Consistently, our results also indicate that a positive expression of cell-surface CSC markers, including CD24, CD133, CD44s, CD44V6, and CD44V9, as well as cytosolic ALDH1 marker, can strongly predict the important clinicopathological parameters along with worse prognostic outcomes in GC cases. Importantly, considering that the related parameters do not overlap, the detection of a combined panel of CSC markers is likely to provide a more precise predictive potential for GC patients. However, CD44s was the only CSC marker linked to tumor differentiation, suggesting involving of CD44s in malignant progression of GC. In addition, data regarding the expression of Lgr-5 showed no association with clinicopathological and prognostic values of patients with the disease. Moreover, a few references exist concerning the link between the expression of CD44V9 and GC clinical outcomes that our results indicate no association between its expression and poor OS and CSS/DSS.

The other specific stemness-related marker types in gastric cancer are transcription factors (TFs) implicated in self-renewal and pluripotency. Key stem cell TFs such as Sox-2, Oct-4, and Nanog have been demonstrated to be overexpressed in CSCs [94]. Our results suggest that, while a high expression of Oct-4 is associated with clinicopathological features that can reduce the OS and DFS/RFS of GC patients, overexpression of Nanog has no association with prognostic and clinicopathological values of patients. The results of a research by Li et al. [49] were also in agreement with our findings, suggesting that Oct-4 might be a more useful prognostic factor for relapse or distant metastasis after operation compared with Nanog and Sox-2. In GC, Sox-2 function is still accompanied by some controversies; some studies reported the link of its overexpression with a more aggressive feature, worse prognosis, and chemoresistance [48, 95], while some other studies have demonstrated the opposite [49]. We found a positive relationship between Sox-2 overexpression and lymph node metastasis as well as OS, but not with DFS/RFS. Bmi-1 is another TF that has been found to importantly regulate the self-renewal capacity of both normal and tumor stem cells [96]. It has been demonstrated that Bmi-1 overexpression might considerably associate with a worse OS in breast cancer cases [97]. However, in our sub-analysis, Bmi-1 did not affect the OS of GC cases. Instantly, lymph node metastasis, TNM stage, and worse CSS/DSS in GC patients were associated with the overexpression of Bmi-1, representing the role of Bmi-1 in the death resulted from GC.

Additionally, many intracellular signaling pathways have been cleared to be involved in the regulation of CSCs [93]. The sonic hedgehog (Shh) signaling cascade is one of these pathways that is occurred through the binding of Shh ligands to transmembrane receptor Patched 1 (PTCH1), which allows the dissociation and conformational change of Smoothened (Smo) receptor, eventually resulting in the activation of three zinc finger Gli transcription factors (Gli-1, Gli-2, and Gli-3) [98]. Gli-1 appears to play a pivotal role in the maintenance of tumor cells with stemness characteristics. In GC, Gli-1 expression has been reported to be positively linked to a more aggressive tumor phenotype [79]. Similarly, our analysis indicates that Gli-1 overexpression not only promotes higher TNM stage and lymph node metastasis strongly but also reduces OS and DFS/RFS in GC patients. On the other hand, the expression of Shh has also been found to contribute to epithelial-to-mesenchymal transition (EMT) in pancreatic adenocarcinoma cell lines [99] as well as lymphatic metastasis in bladder cancer [100]. However, our analyses display that overexpression of Shh is not associated with clinicopathological values and OS in GC patients. Nevertheless, based on only one study [23], its high expression was linked to a poor DFS/RFS in GC patients.

However, our large meta-analysis sheds light on the clinicopathological and prognostic roles of gastric CSC markers; there are several potential limitations that must be considered as well as some results that should be interpreted with caution. Firstly, this meta-analysis was not performed based on the randomized controlled studies with a prospective design, which would have made this study more susceptible to information and selection biases. Secondly, the cut-off values defining CSC markers overexpression in the eligible studies were not based on a universal standard, possibly affecting the findings of this meta-analysis. Third, since most of the included articles in the current study were performed in Asian countries, a potential population selection bias may be produced. Fourth, the present meta-analysis was restricted to the articles published in English, which might be along with selection bias. Fifth, for papers without providing HR with 95% CI directly, we estimated the HRs through Kaplan–Meier curves, which possibly reduces the credibility of the findings. Sixth, partially high heterogeneity was identified in most analyses. Differences in research methodology and race might affect the heterogeneity. Finally, the publication bias was detected for OS and the overexpression of overall CSC markers, therefore likely reducing the reliability of the association between CSCs and worse prognosis. The majority of the studies prefer to selectively publish positive findings, potentially resulting in publication bias. Considering all these limitations, further multicenter prospective investigations on the basis of the standardized methodology are needed to validate the potential of the gastric CSC markers in the prediction of patients’ outcomes.

## Conclusion

In conclusion, findings of our comprehensive meta-analysis reveal a notable role of CSC markers, including cell surface markers, TFs, and components of Shh signaling pathway, in predicting poor clinical outcomes of patients with GC. Previous meta-analyses evaluated only some of these associations and cleared that some CSC markers have prognostic significance for OS of GC patients [101, 102]. However, to the best of our knowledge, this is the first meta-analysis that exclusively included 13 CSC markers with a large sample size, which made the findings more robust and powerful, and on the other side, systematically assessed the clinicopathological and prognostic values of CSC markers, overall and individually, among gastric cancer patients. Although all of the detected CSC markers are not predictors of worse outcomes, most can potentially be known as the prognostic biomarkers. By analyzing the eligible studies, it was cleared that Gli-1, Oct-4, CD44s, CD44V6, and CD133 have strong prognostic values. Our meta-analysis suggests applying a combined panel of CSC markers overexpression for the prediction of gastric cancer patients OS, DFS/RFS, and CSS/DSS and the stratification of different gastric cancer patients. However, due to some certain limitations, various analyses showed relatively inconsistent results, of which careful selection of CSC markers and the standardized methodology are possibly considered as the fundamental ones to optimize the accuracy of CSC markers as prognostic and predictive clinical factors in gastric cancer.

## Supplementary Information

**Additional file 1: Table S1.** Search strategy and syntax in different databases based on the expression of cancer stem cell markers in gastric cancer.

## Data Availability

Not applicable.

## Abbreviations

GC: Gastric cancer
CSC: Cancer stem cell
HRs: Hazard ratios
ORs: Odds ratios
CIs: Confidence intervals
IHC: Immunohistochemistry
OS: Overall survival
DFS: Disease-free survival
CSS: Cancer-specific survival
Shh: Sonic hedgehog
TFs: Transcription factors
NOS: Newcastle*–*Ottawa Scale
PTCH1: Patched 1 receptor
Smo: Smoothened receptor
EMT: Epithelial-to-mesenchymal transition
RFS: Relapse-free survival
DSS: Disease-specific survival
