# Supplementary material for: Clinical and prognostic significances of cancer stem cell markers in gastric cancer patients: a systematic review and meta-analysis
Source: Cancer Cell Int. 2021 Feb 27;21:139. doi: 10.1186/s12935-021-01840-z (PMC7912890; doi:10.1186/s12935-021-01840-z)
Supplement: Supplementary file 1 — Additional file 1: Table S1. Search strategy and syntax in different databases based on the expression of cancer stem cell markers in gastric cancer. [file 12935_2021_1840_MOESM1_ESM.docx]

Table S1: search strategy and syntax in different database based on gastric cancer stem cell markers expression in cancer

| Search strategy | | No. of Papers |
| --- | --- | --- |
|  |  | October 16, 2020 |
| PubMed | ("Neoplasms"[Mesh] OR Neoplasm*[Title/Abstract] OR Cancer*[Title/Abstract] OR tumor*[Title/Abstract] OR malignancy*[Title/Abstract] OR malignant*[Title/Abstract] OR carcinoma*[Title/Abstract]) AND ("Stomach"[Mesh] OR Stomach*[Title/Abstract] OR Gastric*[Title/Abstract]) AND ("Neoplastic Stem Cells"[Mesh] OR Neoplastic Stem Cell*[Title/Abstract] OR Tumor Stem Cell*[Title/Abstract] OR Tumor Initiating Cell*[Title/Abstract] OR Cancer Stem Cell*[Title/Abstract] OR Neoplastic colony-forming unit*[Title/Abstract] OR csc*[Title/Abstract]) AND ("Biomarkers, Tumor"[Mesh] OR "Biomarkers"[Mesh] OR biomarker*[Title/Abstract] OR marker*[Title/Abstract] OR "Antigens, Surface"[Mesh] OR surface antigen*[Title/Abstract] or "Prognosis"[Mesh] OR Prognosis*[Title/Abstract] OR Prognostic*[Title/Abstract] OR "Diagnosis"[Mesh] OR Diagnosis*[Title/Abstract] OR Diagnostic*[Title/Abstract] OR screening*[Title/Abstract] OR detection*[Title/Abstract]) Filters: English | 466 |
| Embase | ('neoplasm':ab,ti OR 'cancer':ab,ti OR 'tumor':ab,ti OR 'malignancy':ab,ti OR 'malignant':ab,ti OR 'carcinoma':ab,ti) AND ('gastric':ab,ti OR 'stomach':ab,ti) AND ('neoplastic stem cell':ab,ti OR 'tumor stem cell':ab,ti OR 'tumor initiating cell':ab,ti OR 'cancer stem cell':ab,ti OR 'neoplastic colony-forming unit':ab,ti OR csc:ab,ti) AND ('biomarker':ab,ti OR 'marker':ab,ti OR 'surface antigen':ab,ti OR 'prognosis':ab,ti OR 'prognostic':ab,ti OR 'diagnosis':ab,ti OR 'diagnostic':ab,ti OR 'screening':ab,ti OR 'detection':ab,ti) AND [english]/lim | 298 |
| Scopus | ‎( TITLE-ABS-KEY ( neoplasm )  OR  TITLE-ABS-KEY ( cancer )  OR  TITLE-ABS-KEY ( tumor )  OR  TITLE-ABS-KEY ( malignancy )  OR  TITLE-ABS-KEY ( malignant )  OR  TITLE-ABS-KEY ( carcinoma ) )  AND  ( TITLE-ABS-KEY ( stomach )  OR  TITLE-ABS-KEY ( gastric ) )  AND  ( TITLE-ABS-KEY ( neoplastic  AND stem  AND cell )  OR  TITLE-ABS- KEY ( tumor  AND stem  AND cell )  OR  TITLE-ABS-KEY ( tumor  AND initiating  AND cell )  OR  TITLE-ABS-KEY ( cancer  AND stem  AND cell )  OR  TITLE-ABS-KEY ( neoplastic  AND colony-forming  AND unit )  OR  TITLE-ABS-KEY ( csc ) )  AND  ( TITLE-ABS-KEY ( biomarker )  OR  TITLE-ABS-KEY ( marker )  OR  TITLE-ABS-KEY ( surface  AND antigen )  OR  TITLE-ABS-KEY ( prognosis )  OR  TITLE-ABS-KEY ( prognostic )  OR  TITLE-ABS-KEY ( diagnosis )  OR  TITLE-ABS-KEY ( diagnostic )  OR  TITLE-ABS-KEY ( screening )  OR  TITLE-ABS-KEY ( detection ) )  AND  ( LIMIT-TO ( LANGUAGE ,  "English" ) ) | 2105 |
| Web of science | ((TS=(neoplasm) OR TS=(cancer) OR TS=(tumor) OR TS=(malignancy) OR TS=(malignant) OR TS=(carcinoma)) AND (TS=(stomach) OR TS=(gastric)) AND (TS=(neoplastic stem cell) OR TS=(tumor stem cell) OR TS=(tumor initiating cell) OR TS=(cancer stem cell) OR TS=( neoplastic colony-forming unit) OR TS=(csc)) AND (TS=(biomarker) OR TS=(marker) OR TS=(surface antigen) OR TS=(Prognosis) OR TS=(Prognostic) OR TS=(Diagnosis) OR TS=( Diagnostic) OR TS=(screening) OR TS=(detection))) *AND*LANGUAGE: (English) | 1556 |
